# Synthesis of transition metal doped lanthanum silicate oxyapatites by a facile co-precipitation method and their evaluation as solid oxide fuel cell electrolytes†

**DOI:** 10.1039/d2ra07088j

**Published:** 2023-04-19

**Authors:** Henri Joel Mbah Ngantchou, Rizwan Raza, Edwin Akongnwi Nforna, John Lambi Ngolui, Tauqir A. Sherazi

**Affiliations:** a Department of Chemistry, COMSATS University Islamabad, Abbottabad Campus Abbottabad 22060 Pakistan sherazi@cuiatd.edu.pk; b Department of Chemistry, Faculty of Sciences, University of Douala P.O Box 24157 Douala Cameroon; c Department of Physics, COMSATS University Islamabad, Lahore Campus Lahore 54000 Pakistan razahussaini786@gmail.com; d Department of Fundamental Science, Higher Technical Teacher Training College, The University of Bamenda P.O. Box 39 Bambili Cameroon; e Chemistry Department, Higher Teacher Training College, University of Yaoundé I Yaoundé 47 Cameroon

## Abstract

Transition metal doped apatite La_10_Si_6−*x*_Co_*x*_O_27−*δ*_ (*x* = 0.0; 0.2; 0.8) and La_10_Si_5.2_Co_0.4_Ni_0.4_O_27−*δ*_ are synthesized by co-precipitation method followed by sintering. The precursor precipitates and apatite products are characterized by XRD, FTIR, TGA/DTA, Raman Spectroscopy, SEM-EDX and electrochemical impedance spectroscopy. The presence of apatite phase with hexagonal structure is confirmed through the XRD results. The conductivity measurements of the samples sintered at 1000 °C show that the ionic conductivity increases with increasing content of Co^2+^ doping into apatite that is further increased by co-doping of Ni^2+^. The Co doped apatite (La_10_Si_5.2_Co_0.8_O_27−*δ*_) exhibited conductivity of 1.46 × 10^−3^ S cm^−1^ while Co–Ni co-doped sample (La_10_Si_5.2_Co_0.4_Ni_0.4_O_27−*δ*_) exhibited highest conductivity of 1.48 × 10^−3^ S cm^−1^. The maximum power density achieved is also for Co, Ni co-doped sample *i.e.*, 0.65 W cm^−2^ at 600 °C. The results represented show that Co and Ni enhances the SOFC performance of apatite and makes it potential electrolyte candidate for solid oxide fuel cell application.

## Abstract 2

The reported results represented that Co and Ni enhances the SOFC performance. It is potential electrolyte candidate for solid oxide fuel cell.

## Graphical abstract

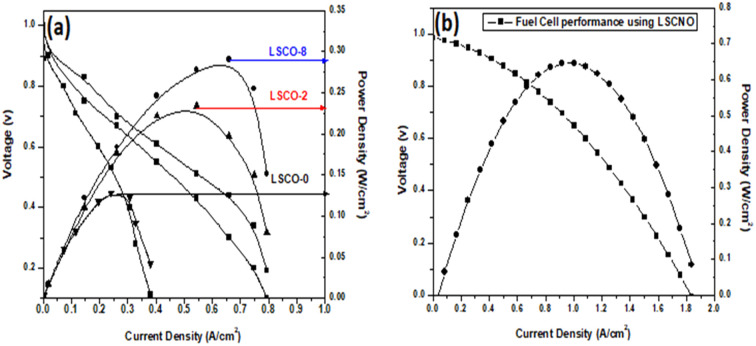

## 1. Introduction

The advancement of industrial technology and rapid growth of world population has resulted in a high demand for energy. The increase in the demand for energy has generated a great interest to develop the devices which will provide cheap, renewable energy. One of such devices is the solid oxide fuel cells (SOFCs), an electrochemical device that is an efficient source of electrical energy to comply with the future energy demand ^1–3^. The SOFC is made up of the electrodes, electrolyte, and interconnect materials all in the solid state. The electrolyte materials for SOFCs must have a wide range of characteristics including negligible electronic conductivity, high ionic conductivity (oxide ion or proton), high ion transport numbers in a wide range of oxygen partial pressures, and chemical stability at high temperatures under partially reducing/oxidizing conditions.^4^ The stabilized zirconia, which has the fluorite structure, was first used in SOFC in 1937, although it had previously been employed as the electrolyte in the Nerst-lamp ^5^. The most widely used stabilizers are yttrium (YSZ) or scandium (SSZ).^6^ The conductivities of these solid solutions increase with the degree of substitution to an optimum 8% for Y_2_O_3_ and 11% for Sc_2_O_3_,^7^ and this maximum conductivity is reached at a degree of substitution close to the minimum that stabilizes the cubic fluorite phase.^8^ The scandium stabilized zirconia (SSZ) experiences higher ionic conductivities than Yttrium stabilized zirconia (YSZ) but the SSZ is relatively much expensive.^9^ Consequently, YSZ has become the electrolyte most widely used in SOFCs that shows good properties as an electrolyte material. However, the major drawback is its high working temperatures (800–1000 °C) depending on the thickness of the electrolyte that is required to achieve sufficient ionic conductivity. This high temperature requirement limits the use of other cell components to have high temperature resistance in order to be compatible with the electrolyte material and also high temperature sealing must be used, which causes additional cost increase of the SOFC system. One of the focus areas of SOFCs research is the development of the electrolyte material that would operate at relatively lower temperatures. In this research, we exploit the lanthanum silicate apatites as electrolyte material for SOFCs. Nakayama *et. al*., were the first to discover oxyapatite-structured materials with the general formula RE_9.33+*x*_Si_6_O_26+1.5*x*_ (where RE represents rare earth elements) ^10^. Since then, they have attracted particular attention because of their high oxide ion conductivity and low activation energies. An interesting aspect in materials science is the modification of properties of materials through doping. This is the approach utilized in this research to seek superior ionic conductivity of lanthanum silicate oxyapatites. These studies showed that non-stoichiometry in the form of either cationic vacancies or excess oxygen is required to achieve good oxide ion conductivities ^11,12^. Different types of doping on the lanthanum site or on the silicon site, or on the both have been studied. Some studies of the Co-doped lanthanum silicate oxyapatite systems, La_10_Si_6−*x*_Co_*x*_O_27−*x*/2_, synthesized by the sol–gel method have been reported by Qingle *et. al.*^13^ The results showed that as the Co level increased, an initial increase in conductivity was observed, reaching a maximum value for *x* = 0.8 (La_10_Si_5.2_Co_0.8_O_26.6_). Higher Co^3+^ concentrations (0.8 < *x* < 1.5) results in a decrease in the conductivity of La_10_Si_6−*x*_Co_*x*_O_27−*x*/2_ because of the lower concentration of interstitial oxide ions and greater stoichiometry. Therefore, excess Co^3+^ dopant is unfavorable for high oxide conductivity. The doping results as shown above indicate clearly that oxygen over-stoichiometry is responsible for the good oxide-ion conductivity.

The level and doping site both are important to improve the conductivity. In general, it has been shown that the ionic conductivity decreases when doping is carried out at the lanthanum site, such that the conductivity of La_8.67_SrSi_6_O_26_ is found to be 8.3 × 10^−5^ S cm^−1^ at 500 °C that is lower than 1.1 × 10^−4^ S cm^−1^ for La_9.33_Si_6_O_26_ (ref. 14) because of decrease in the number of cationic vacancies.

Different synthesis methods have been reported for the nanocrystalline lanthanum silicate powders, distinguished according to preparation temperature as solid state reaction and solution synthesis.^15^ The high preparation temperature methods have the common problem that precursors do not properly mix on a large scale.

Low-temperature preparation methods have greatly overcome the problem of precursor contact; however, side products are still detected in the compounds obtained with these methods. Recently, some authors have^16,22^ reported a facile co-precipitation method to synthesize nano-sized LSO powders, where La(NO_3_)_3_·6H_2_O and tetraethyl orthosilicate (TEOS) are dissolved in water and ethanol to precipitate well-mixed precursors under dilute ammonia solution. Qingle *et. al*.^13^ used the sol–gel method for the synthesis of La_10_Si_6−*x*_Co_*x*_O_27−*x*/2_, with starting Co^3+^ ions. While the present work reports the modified co-precipitation method based on a single source metal–organic precursor, that is, the metal octanoate to synthesize fine powders of apatite La_10_Co_*x*_Si_6−*x*_O_27−*δ*_, where *x* is maintained as 0.0, 0.2 and 0.8. In addition to doping with Co^2+^, co-doping with Co and Ni in the sample (La_10_Si_5.2_Co_0.4_Ni_0.4_O_27−*δ*_) is also synthesized in this work, which have not yet been reported. The synthesized samples were calcined at different temperatures and characterized by TG/DTA, XRD, FT-IR, Raman spectra and SEM-EDX to identify the pure phase formation and the morphologies. The effect of partial substitution of silicon by divalent cobalt, Co^2+^ and Ni^2+^ on the structure and grain morphology are examined. Electrical conductivity properties as a function of temperature under air by electrochemical impedance spectroscopy are investigated to determine the application of material as an electrolyte for SOFC.

## 2. Experimental method

### 2.1. Synthesis of apatites

La(NO_3_)_3_·6H_2_O, Co(NO_3_)_2_·6H_2_O, Ni(NO_3_)·6H_2_O of analytical grade were purchased from Sigma-Aldrich. The tetraethyl orthosilane (98%, Acros Organics), octanoic acid (99.99%, Merck), sodium hydroxide (98%, BIOCHEM Chemopharma United Kingdom), absolute ethanol (Baker) were used as received without further purification. De-ionized (DI) water was used throughout the study.

The cobalt-doped lanthanum silicate of the general formula La_10_Si_6−*x*_Co_*x*_O_27−*δ*_ (where *x* = 0.0; 0.2; and 0.8) were synthesized in this work. For each composition, the required stoichiometric amounts of lanthanum nitrate, cobalt(ii) nitrate, nickel nitrate and tetraethyl orthosilicate (TEOS) were dissolved in DI water along with continuous stirring to obtain a homogeneous solution. The sodium octanoate was used as precipitating agent that was prepared by mixing the solutions of octanoic acid and sodium hydroxide. The solutions containing the metal salts (La^3+^, Co^2+^, Ni^2+^and Si^4+^) and sodium octanoate were mixed with continuous stirring for about 1 hour at room temperature. The resulting precipitates of precursor (La–Si–Co or La–Si–Co–Ni octanoate) were then filtered to remove the NaNO_3_, followed by washing with ethanol and drying in an oven for 12 hours at 80 °C. The as-prepared precursor powder was sintered for 4 h in air at different temperatures in a tubular furnace in air (heating rate 10 °C min^−1^).

The cobalt-doped lanthanum silicate of the general formula La_10_Si_6−*x*_Co_*x*_O_27−*δ*_ with *x* = 0.0; 0.2 and 0.8 are assigned the codes LSCO-0, LSCO-2 and LSCO-8 respectively, while the various precursors from which they are derived are labelled with the codes P-00, P-02, P-08. The compound La_10_Si_5.2_Co_0.4_Ni_0.4_O_27−*δ*_ is assigned the code LSCNO and its precursor PCN-04.

### 2.2. Characterizations

In order to determine the calcination temperature, the precursors were analysed by thermogravimetric analysis (TGA). The TGA measurements were performed using DTA 1600 TA Instruments, USA, under oxygen flow rate of 100 ml min^−1^, up to a temperature of 900 °C, with a heating rate of 20 °C min^−1^. The crystalline phases were identified by X-ray diffraction (XRD) analysis (Bruker AXS D8 Focus) using Co Kα radiation (*λ* = 1.78901 Å). The angular scan range is 20°–100° and the scan increment is 0.02° per step for all samples. The microstructure characterization of the obtained powders is carried out by scanning electron microscope (JSM5800LV, JEOL, Japan) combined with an EDX holder. The FTIR spectra of samples were recorded using IR Fourier spectrometer Nicolet 6700 equipped with Smart Orbit™ diamond ATR accessory (Thermo Scientific, USA). The FTIR Spectra were obtained in the range of 400–4000 cm^−1^. Electrical conductivity measurements as a function of temperature were performed by the complex impedance spectroscopy technique, using a Solartron 1260A frequency response analyser (FRA) over the frequency range 5 Hz to 15 MHz, under static air. The measurements were made in open circuit and with an applied AC voltage of 100 mV. Impedance diagrams are recorded from 450 to 750 °C with thermal steps of about 25 °C and settling times of 20 minutes. The fuel cell performance were performed with hydrogen fuel. The measurments were performed at 600 °C.

## 3. Results and discussion

### 3.1. Characterization of precursors

#### 3.1.1. Analysis by Fourier transform infrared spectroscopy (FTIR)

**Fig. 1:**
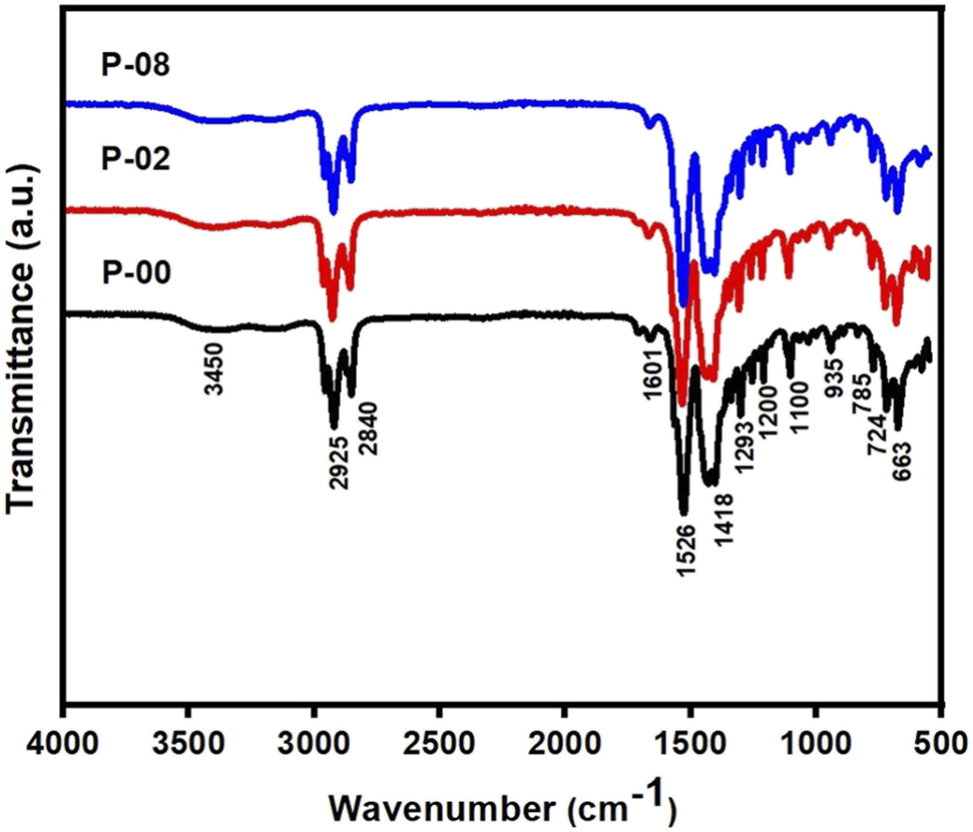
Fourier transform infrared (FTIR) spectra of precursors of the samples LSCO-0, LSCO-2, and LSCO-8.

The FTIR spectra of the octanoate precursors of LSCO-0, LSCO-2 and LSCO-8 samples are presented in Fig. 1. It can be observed that all the spectra are similar, *i.e.* that all the peaks emerge at practically the same positions and therefore representing the presence of same functional group. These FTIR spectra allow us to deduce that cobalt doping has no influence on the functional groups in the samples, which is normal because the addition of cobalt does not modify the chemical nature of the existing ligands in the precursor compound.

The absence of vibration of the free carbonyl (C=O) around 1720 cm^−1^ and 1740 cm^−1^ is an indication that the carboxylate group of the octanoate has fully engaged in the coordination bond. From Fig. 1, the intense peaks at 2925 and 2840 cm^−1^ are typical of symmetric and asymmetric stretching vibrations of C–H in the –CH_3_ and –CH_2_ groups respectively.^17^ The peaks at 1418 cm^−1^ and 1200 cm^−1^ are attributed to the scissors deformation and wagging vibrations of C–H, respectively. Although the characteristic band of the C=O group of carbonyl compounds and carboxylic acids is missing due to the coordination bond formation but the two CO groups in the octanoate ligands give rise to asymmetric and symmetric frequencies at 1601 and 1526 cm^−1^, respectively.^17^

This is evidence of the presence of carboxylate groups in the samples. It can be demonstrated that the value of the separation between the asymmetric and symmetric stretching of COO, *i.e.*, Δ*ν* (υCOOasy – υCOOsym) can be used to determine the binding mode between the carboxylate group (COO) of the ligand and the metal. The value of Δ*ν* below 140 cm^−1^ is attributable to the bidentate chelating mode while Δ*ν* between 140 cm^−1^ and 200 cm^−1^ is attributed to the bridged bidentate binding mode and Δ*ν* above 200 cm^−1^ is attributed to monodentate binding mode.^18^ From the spectra, it is observed that the octanoate ligands exhibit bidentate chelating effect to the metal in all the compounds. These results are similar to those reported in the literature for similar compounds.^19^ These results confirm the presence of the expected functional groups in the precursors: P-00, P-02, and P-08.

#### 3.1.2. Thermal behavior and phase formation of the La_10_Si_5.8_Co_0.2_O_27−*δ*_ precursor

**Fig. 2:**
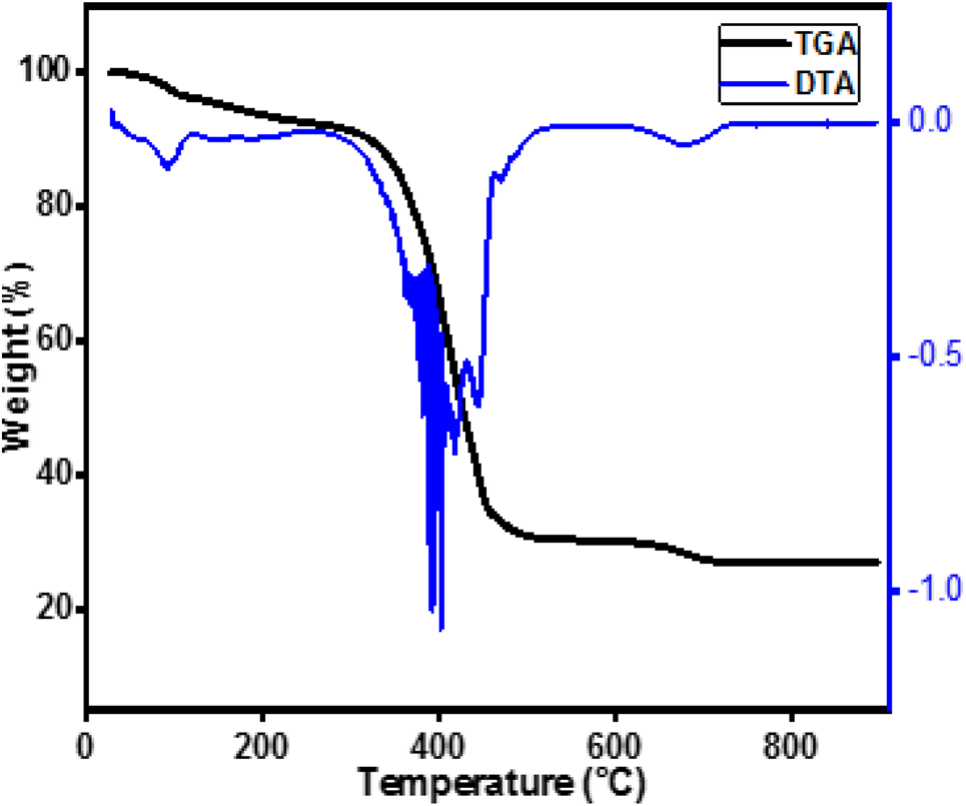
Thermogravimetric curve and DTA of sample P-02 synthesized by co-precipitation method.

Thermogravimetric analysis (TGA) of the precursor of the La_10_Si_5.8_Co_0.2_O_27−*δ*_ sample was performed to determine its decomposition temperature as plotted in Fig. 2. The differential thermal analysis curve showed that there are three major regions of weight loss. The first region falls around 100 °C that is due to removal of free and some of bound water. The second region falls between 350 and 450 °C that is attributed to the decomposition of organic part present in the sample. This temperature range is responsible for decomposition of single metal octanoates.^20^ There is a small decomposition around 700 °C. The total weight loss from 100 °C and 700 °C is about 73% that is mainly corresponds to the loss of the organic fraction (theoretical percentage 76%). However, there is no further weight loss observed at temperature above 800 °C, which confirms the absence of any further decomposition. The residue left after complete decomposition was 26.91% that is quite close to the theoretical value of 23.54%, which corresponds to the mixed oxide La_10_Si_6−*x*_Co_*x*_O_27−*δ*_. From the TGA curve, it is considered that 800 °C is a suitable temperature to treat the precursor for the synthesis of apatites.

### 3.2. Analysis of the apatites

#### 3.2.1. Infrared spectra

The vibration frequencies in the infrared region are fundamental in the determination of crystalline structures.^21^ IR analysis of synthesized samples is important both for the control of the reaction process and the properties of materials obtained. The FTIR spectra in the region 500–4000 cm^−1^, of the mixed metal oxide of the products (LSCO-0, LSCO-2, and LSCO-8) obtained after the decomposition at 1000 °C are presented in ESI† Fig. 1. As the spectra indicate, most of the bands such as for the carboxylate (OCO) group (1601 and 1526 cm^−1^) and aliphatic carbon–hydrogen (–CH) groups (2925 and 2840 cm^−1^) are now absent. The careful observation of the IR spectra indicates characteristic absorption bands of the different metal oxides. The bands at about 976 and 928 cm^−1^ can be attributed to the stretching vibrations of the Si–O–Si and Si–O bonds, respectively. The La–O bond is observed around 545 cm^−1^. In general, the higher frequency band of about 627 cm^−1^ corresponds to the M–O stretching vibration mode that may include the stretching vibration frequencies for La–O, Ni–O or Co–O bonds. However, the relatively strong bands at 1463–1360 cm^−1^ may be due to the stretching vibrations in NO_3_^−^, which may arise from a small fraction of nitrate from the reactants trapped in the crystal lattice. The observed IR band at 3600 cm^−1^ may be due to the stretching vibration modes of the O–H bonds in water molecules or in surface silanol bonds, Si–OH (Si bonded to water molecules).

#### 3.2.2. Crystalline structure analysis using XRD

**Fig. 3:**
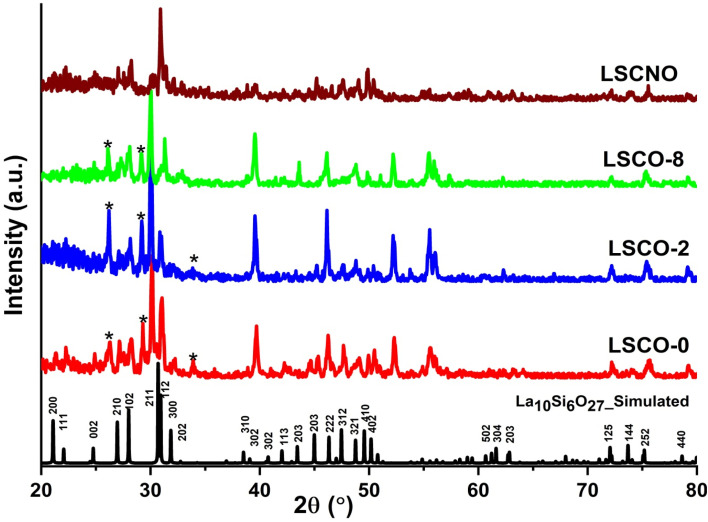
XRD patterns of simulated La_10_Si_6_O_27_ (ICDD Card No. 053-0291), La_10_Si_6−*x*_Co_*x*_O_27−*δ*_ and La_10_Si_5.2_Co_0.4_Ni_0.4_O_27−*δ*_ calcined at 1000 °C.

The XRD patterns of La_10_Si_6−*x*_Co_*x*_O_27−*δ*_ (*x* = 0.0, 0.2, 0.8) and La_10_Si_5.2_Co_0.4_Ni_0.4_O_27−*δ*_ powders calcined at 1000 °C for 4 hours are presented in Fig. 3. The XRD patterns are indexed on the standard ICCD card No. 053-0291. All the experimental peaks matched with the standard indicating oxy-apatite phase. The synthesized samples thus exhibit hexagonal structures with space group *P*6_3_/*m*. La_2_O_3_ peaks were absent. Some other peaks marked with an asterisk (*) are observed that might correspond to the apatite phase La_10−*x*_Si_6_O_26+*δ*_, where 0 < *x* < 1 since such peaks appear in the standard pattern of La_9.31_Si_6.24_O_26_ and/or NO_3_^−^ (ICDD card No 40-0234). Other researchers obtained the apatite-type lanthanum silicates from various synthesis methods including co-precipitation, sol–gel and also reported hexagonal structure.^13,22^ However, we observed that the apatite phase was formed at temperatures from 800 °C, which is much lower than the other methods described in the literature.

Lattice parameters and unit cell volume for the hexagonal structure were calculated from the X-ray diffraction data and are presented in Table 1 along with some values from the literature. It is observed that there is a general increase in the unit cell volume and lattice parameters with increasing dopant content. This can be explained by the fact that larger Co^2+^ (0.58 Å) and Ni^2+^ ions (0.55 Å) are substituting smaller Si^4+^ ions (0.26 Å). The expansion of the lattice is evidently seen with the XRD peaks shifting to the left towards lower angles (and higher *d*-spacings). The lattice parameters are very close to those reported in the literature as can be seen in Table 1.^13,16^

**Table 1:** Lattice parameters of La_10_Si_6−*x*_Co_*x*_O_27−*δ*_ and La_10_Si_5.2_Co_0.4_Ni_0.4_O_27−*δ*_ calcined at 1000 °C

Sample code	*a* (A°)	*c* (A°)	*V* (A°)^3^	*D* (nm)	References
LSCO-0	9.56	7.19	569.1	42.11954	This work
	9.7260	7.1884			16
LSCO-2	9.59	7.15	568.7	41.15735	This work
	9.726(2)	7.182(1)	588.3(3)		13
LSCO-8	9.80	7.19	598.0	37.60944	This work
	9.737(1)	7.240(1)	594.5(2)		13
LSCNO	9.81	7.37	614.2	49.29115	This work

The particle size was calculated from the line broadening of the most intense peak using the Scherer formula (*D* = 0.9*λ*/*β* cos *θ*), where *D* is the particle size, *λ* is the wavelength of the radiation, *β* is the full width at half maximum, FWHM and *θ* is the diffraction angle. All the particles are observed to fall in the nanometer range.

#### 3.2.3. Raman spectroscopy

**Fig. 4:**
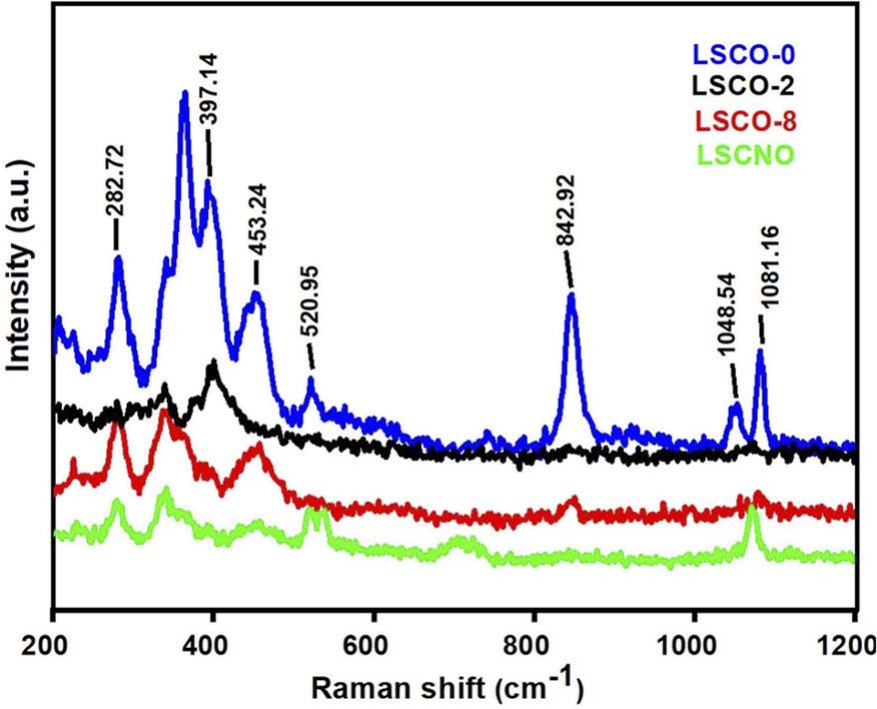
Raman spectrum of the La_10_Si_5.2_Co_0.4_Ni_0.4_O_27−*δ*_ sample, calcined at 1000 °C.

The structure of apatite is composed of a quasi-compact arrangement of tetrahedral SiO_4_ groups. As the Si–O bonds in the tetrahedra are stronger than the La–O bonds,^23^ the vibrational spectrum of the apatite will be divided into internal vibrations due to strong bonds in the SiO_4_, and external vibrations that is linked to the rest of the structure. The internal vibration modes of the isolated SiO_4_ unit are divided into four vibration modes including a symmetrical elongation mode of frequency *υ*_1_, a symmetrical mode of angular deformation *υ*_2_, an asymmetric mode of elongation *υ*_3_, and an asymmetric mode of angular deformation *υ*_4_. Vibration modes *υ*_1_ and *υ*_2_ are more intense in Raman spectroscopy.^23,24^Fig. 4 shows the Raman spectrum of the La_10_Si_6−*x*_Co_*x*_O_27−*δ*_, and La_10_Si_5.2_Co_0.4_Ni_0.4_O_27−*δ*_ powder sample calcined at 1000 °C. In Fig. 4, an intense band can be observed at 362.04 cm^−1^ and 397.14 cm^−1^, which is attributed to the symmetric deformation mode (*ν*_2_) of the SiO_4_ tetrahedral unit. The Raman bands observed at 1048.54 and 1081.16 cm^−1^ correspond to the asymmetric stretching modes (*ν*_3_) of the tetrahedral SiO_4_ unit molecule. In addition, another such intense band at 842.92 cm^−1^ is attributed to the symmetric bending mode (*ν*_1_) and the band at 453.24 and 520.95 cm^−1^ is attributed to the asymmetric bending mode (*ν*_4_) of the SiO_4_ tetrahedral unit. The Raman band observed at 282.72 cm^−1^, and all other bands below it correspond to the La–O_(SiO4)_ vibration.

#### 3.2.4. Microstructure and elemental analysis of prepared samples

**Fig. 5:**
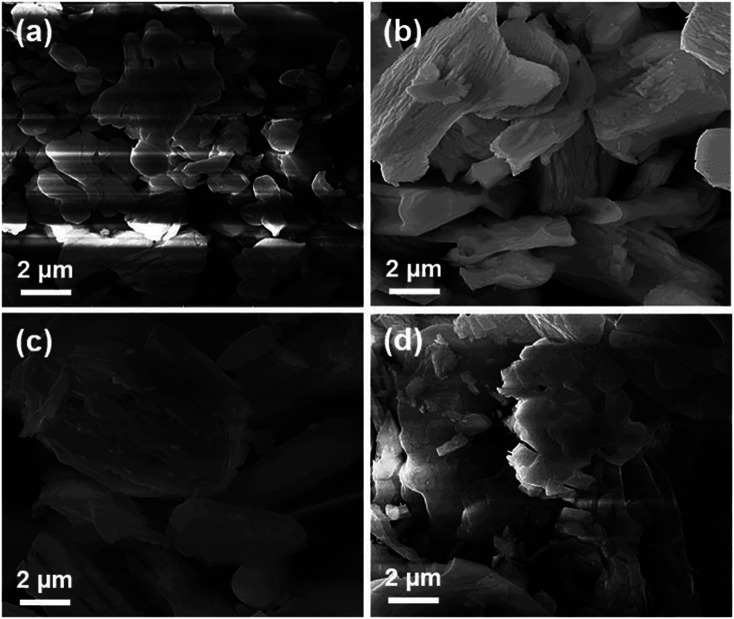
SEM images of La_10_Si_6−*x*_Co_*x*_O_27−*δ*_ calcined at 1000 °C: (a) *x* = 0.0; (b) *x* = 0.2; (c) *x* = 0.8 and (d) La_10_Si_5.2_Co_0.4_Ni_0.4_O_27−*δ*_ calcined at 1000 °C.

In order to understand the density, grain boundaries and especially the phase purity, the SEM technique is used.^25^Fig. 5 shows scanning electron micrographs of all lanthanum silicate apatite samples obtained after calcination at 1000 °C. The SEM microstructures indicate that the particles of the La_10_Si_6−*x*_Co_*x*_O_27−*δ*_ and La_10_Si_5.2_Co_0.4_Ni_0.4_O_27−*δ*_ samples are agglomerated in the form of grains of variable sizes. The average grain size is about 5 μm. The individual particles agglomerated in a definite pattern, forming layered assembly. Each sample looks uniformly distributed, homogeneous and possesses negligible porosity.

**Fig. 6:**
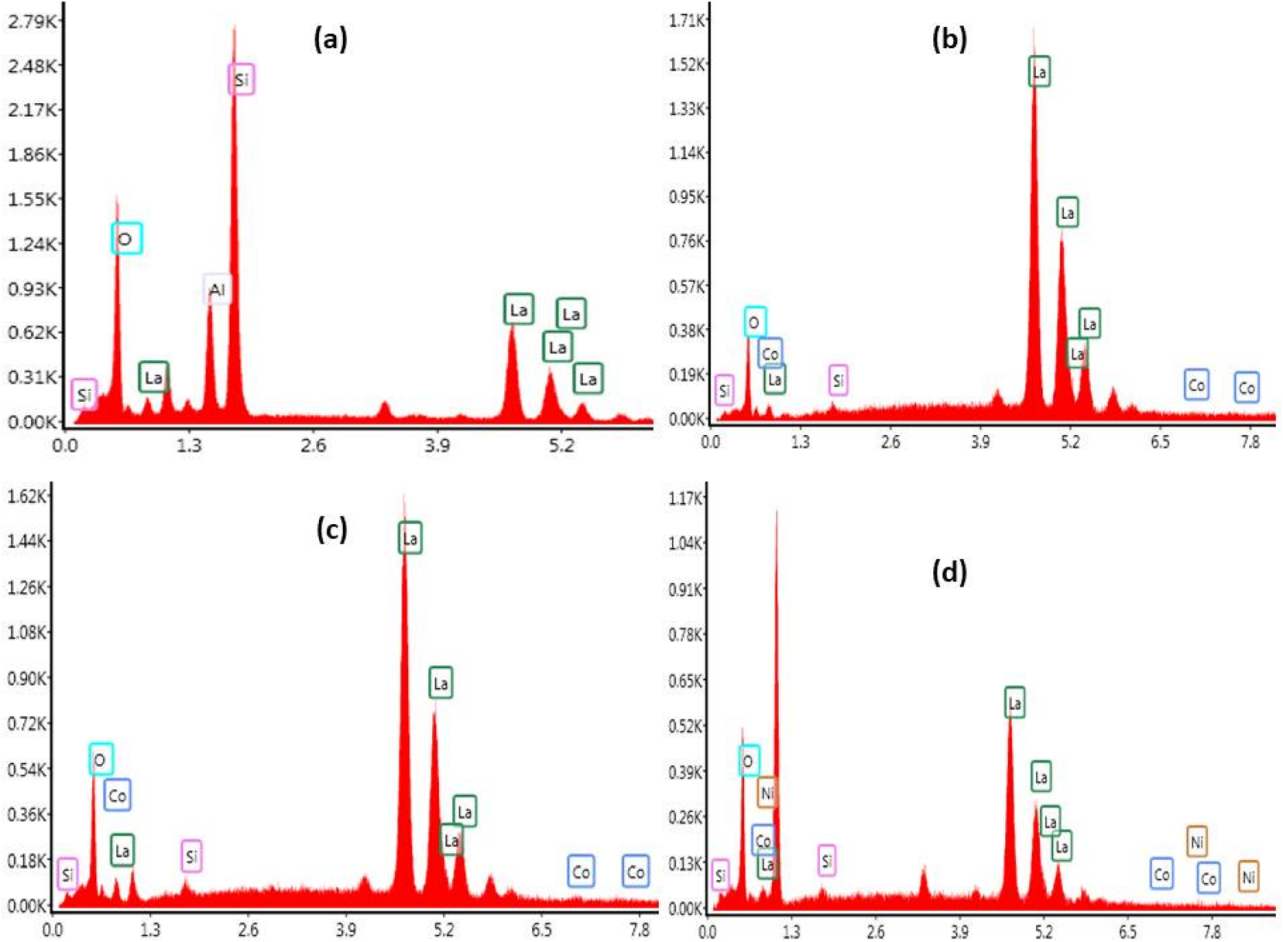
EDX of La_10_Si_6−*x*_Co_*x*_O_27−*δ*_ calcined at 1000 °C: (a) *x* = 0.0; (b) *x* = 0.2; (c) *x* = 0.8 and (d) La_10_Si_5.2_Co_0.4_Ni_0.4_O_27−*δ*_ calcined at 1000 °C.

The SEM was coupled to Energy Dispersive X-ray Spectroscopy (EDX) technique. Fig. 6 shows EDX pattern of La_10_Si_6−*x*_Co_*x*_O_27−*δ*_ and La_10_Si_5.2_Co_0.4_Ni_0.4_O_27−*δ*_. The EDX being a qualitative and a semi-quantitative technique indicated the presence of La, Si and O for the *x* = 0.0 sample while additional Co is also detected for the cobalt doped samples. La_10_Si_5.2_Co_0.4_Ni_0.4_O_27−*δ*_ detected the presence of all the expected elements, that is, La, Si, Co, Ni and O.

#### 3.2.5. Electrical characterization

**Fig. 7:**
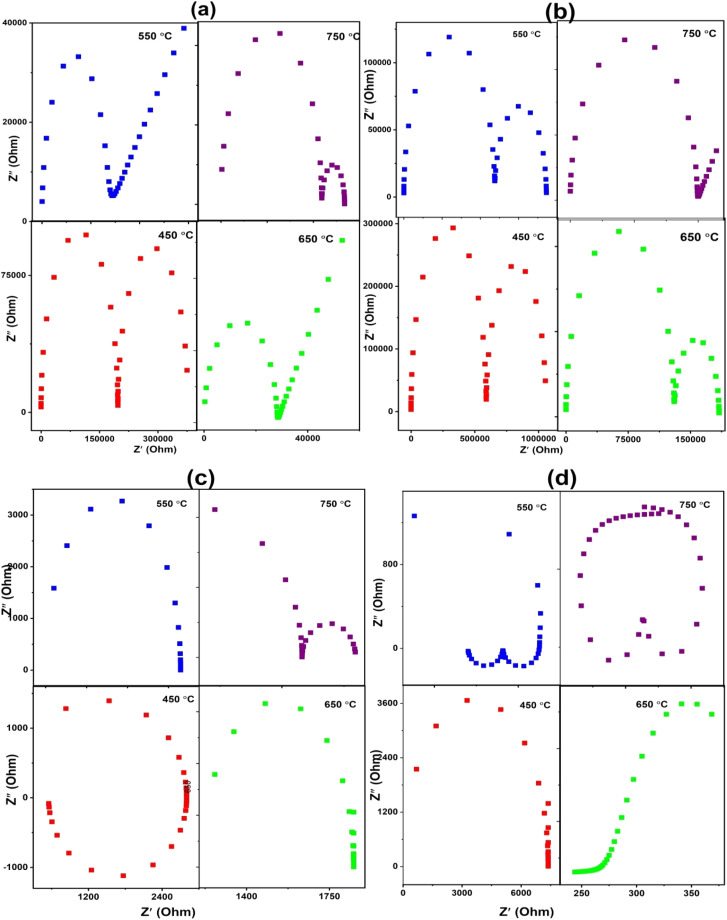
Complex impedance plots of apatite samples (a), LSCO-0, (b) LSCO-2, (c) LSCO-8, and (d) LSCNO-8 measured at different temperatures.

The conductivities of LSCO-0, LSCO-2, LSCO-8 and LSCNO oxyapatites sintered at 1000 °C are investigated by electrochemical impedance spectroscopy in the temperature range of 450–750 °C, Fig. 7 shows the Nyquist plot of the impedance spectroscopy performed on LSCO and LSCNO samples at different temperatures (450 °C, 550 °C, 650 °C and 750 °C). At higher temperature like 750 °C, the spectrum of the compound clearly shows two semicircles with different frequencies corresponding to the bulk resistance (*R*_b_), and the grain boundary resistance (*R*_gb_) respectively.^13,26^ At low temperatures (450 °C and 550 °C), the spectra are composed of two semicircles for LSCO-0 and LSCO-2 which may correspond to the electrode resistance, *R*_el_ and the material's resistance. The bulk resistance, *R*_b_ and the grain boundary resistacne, *R*_gb_ are indistinguishable.^13,26^ For LSCO-8 and LSCNO at low temperature, only one semicircle is observed for the electrode resistance. As the temperature increases, all the samples show two semicircles with different frequencies corresponding to the electrode resistance, *R*_el_ and materials resistance (bulk resistance (*R*_b_) and grain boundary resistance, *R*_gb_). The increase in temperature results in decrease of the semicircles.

**Fig. 8:**
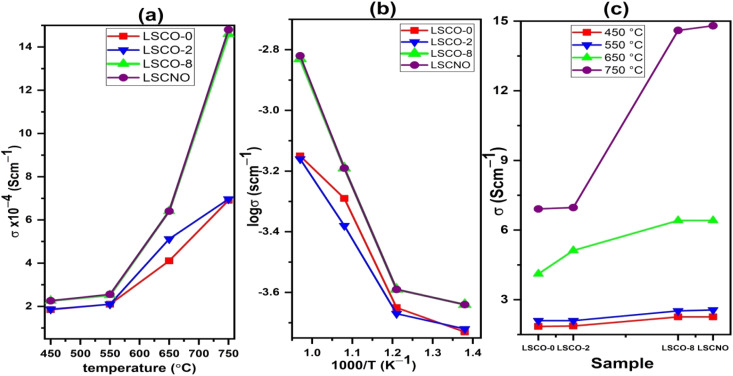
(a) Evolution of conductivity with temperature (b) Arrhenius plot of ionic conductivity and (c) dependence of conductivity with Co and Ni contents.

The total oxygen ion conductivity of the samples operating at different temperatures was calculated using the following equation:

1 *σ* = *L* (*R*·*A*)

where *L* is the thickness (cm) of the pellet, *A* is its surface area (cm^2^), and *R* is the total resistance (Ω) of the sample (grains and grain boundaries). Fig. 8(a) reports the total conductivity of the different compositions carried out in this work at temperatures ranging from 450 to 750 °C. From Fig. 8(a), it is observed that the ionic conductivity gradually increases with temperature and reaches to its maximum value at 750 °C. This is evidence that the ionic diffusion process is a dominant process that is activated by heat.^27^Fig. 8(b) shows the plot of the logarithm *σ*(*T*) *versus* 1000/*T* of the calculated electrical conductivity (*σ*) for different samples sintered at 1000 °C during 4 hours and the data points are fitted to the Arrhenius equation:

2 *σ*(*T*) = *σ*_0_Exp(−*E*_a_/K*T*)

where *σ* is the ionic conductivity (S cm^−1^) of the sample, *T* is the temperature (K), *E*_a_ is the activation energy (eV), *k* is the Boltzmann's constant (8.62 × 10^−5^ eV K^−1^), and *σ*_0_ is the pre-exponential term of Arrhenius' law. The line fitting indicates that the oxide ion diffusion process is thermally activated.^28^

The Arrhenius diagram in Fig. 8(b) shows us a linear evolution of the ionic conductivity. The linear evolution of ionic conductivity indicate that the conductivity is thermally activated, and the activation energy is 0.30, 0.28, 0.39, 0.40 eV for sample LSCO-0, LSCO-2, LSCO-8, and LSCNO, respectively. The values are relatively less in comparison with the literature data (0.5–0.9 eV without impurity phase).^29^

It is also observed that the effect of Co^2+^ doping increases the conductivity of the different materials. This effect is shown in Fig. 8(c). The best value obtained in the case of our work is for *x* = 0.8 at 750 °C with a value of 1.48 × 10^−3^ S cm^−1^. These results are similar to those obtained by Qingle *et al.*^13^ who also showed that the conductivity increased with the doping level of cobalt up to a certain threshold and then decreased beyond *x* = 0.8. The sample that was co-doped with nickel and cobalt, La_10_Si_5.2_Co_0.4_Ni_0.4_O_27−*δ*_ has conductivities at temperatures 550 °C and 750 °C very slightly higher than the *x* = 0.8 sample. However, the conductivity of these sample increases exponentially with temperature increase from 550 °C to 650 °C and then even higher conductivity values achieved at 750 °C. Ni doped lanthanum silicate oxyapatite, La_9.33_Si_6−*x*_Ni_*x*_O_26−*x*_ (*x* = 1.0) showed conductivity of 1.21 × 10^−3^ S cm^−1^ at 700 °C ^30^ while the Co and Ni co-doped sample, La_10_Si_5.2_Co_0.4_Ni_0.4_O_27−*δ*_ in this work showed a higher value of 1.48 × 10^−3^ S cm^−1^ at 750 °C.

The La_10_(SiO_4_)_6_O_3_ compound is made up of isolated SiO_4_ units forming two distinct types of interstices parallel to the c-axis. The smaller insterstice contains La^3+^ cations while the larger one is occupied by La^3+^ and O^2−^ ions.^31^ Conduction is through the interstitial oxide ions. Co doping at the Si sites creates cation vacancies. The oxide ions can hop through these vacant sites,^14^ which results in increase in the ionic conductivity as shown with higher Co doping content.

#### 3.2.6. Fuel cell performance with hydrogen

**Fig. 9:**
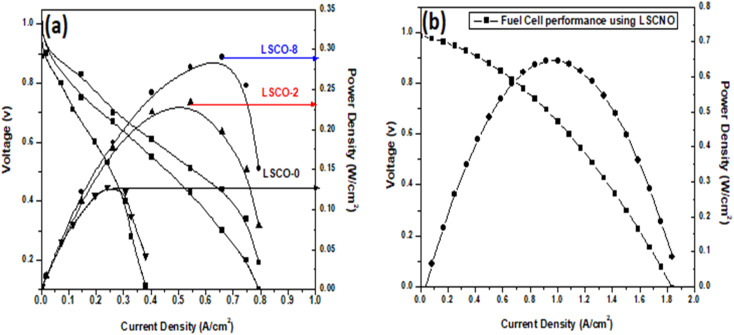
Fuel cell performance of (a) LSCO-0, LSCO-2, LSCO-8, and (b) LSCNO-8 using hydrogen as fuel.

All the prepared electrolyte samples were used to prepared the fuel cells to evaluate their performance as shown in Fig. 9. The testing unit LC-43 from China is used for *I*/*V* measurements at 600 °C. The three apatite samples with and without Co doping; LSCO-O, LSCO-2, LSCO-8 showed the fuel cell performance as 0.290 W cm^−2^, 0.230 W cm^−2^ and 0.130 W cm^−2^, respectively, whereas hydrogen was used as a fuel. The LSCNO experiences the best fuel cell performance such that it exhibited power density of 0.65 W cm^−2^ at 600 °C. Therefore, LSCNO is very suitable electrolyte for solid oxide fuel cell because of its best conductivity and fuel cell performance.

## 4. Conclusion

Samples of La_10_Si_6−*x*_Co_*x*_O_27−*δ*_ (*x* = 0.0; 0.2; 0.8) and La_10_Si_5.2_Co_0.4_Ni_0.4_O_27−*δ*_ were successfully synthesized by the co-precipitation method. Compared to the solid state reaction, the apatite phase was obtained at a lower temperature of 1000 °C with the co-precipitation method. The XRD and Raman spectra confirmed the presence of the apatite phase in the samples. The Co^2+^ doping increases the conductivity of the samples. The co-doping of Co^2+^ and Ni^2+^ in the apatite sample “La_10_Si_5.2_Co_0.4_Ni_0.4_O_27−*δ*_” further improved the conductivity as compared to “La_10_Si_5.2_Co_0.8_O_27−*δ*_ sample, that may be attributed to the minimal difference of ionic radii between Co^2+^ and Ni^2+^ ions. The reported procedure offered the lower temperature sintering without compromising the ionic conductivities. The ionic conductivity for LSCO-8 is achieved as 1.46 × 10^−3^ S cm^−1^ at 750 °C, which is comparable conductivities in the literature at temperatures around 800 °C. These compounds have good ionic conductivities which can have application as electrolyte material in solid oxide fuel cells. The maximum power density with sample LSCNO is 0.65 W cm^−2^ at 600 °C that is much better than the other three samples LSCO-O, LSCO-2, LSCO-8. Therefore, LSCNO is the best potential electrolyte candidate for solid oxide fuel cell owing to its better conductivity and fuel cell performance.

## Conflicts of interest

The authors declare no competing financial interest.

## Supplementary Material

RA-013-D2RA07088J-s001
