# Supplementary material for: Synthesis of transition metal doped lanthanum silicate oxyapatites by a facile co-precipitation method and their evaluation as solid oxide fuel cell electrolytes
Source: RSC Adv. 2023 Apr 19;13(18):12285–94. doi: 10.1039/d2ra07088j (PMC10113921; doi:10.1039/d2ra07088j)
Supplement: RA-013-D2RA07088J-s001 [file RA-013-D2RA07088J-s001.pdf]

Electronic Supplementary Material (ESI) for RSC Advances.
This journal is © The Royal Society of Chemistry 2023

Supplementary figure 1. FTIR absorption spectra of the La10Si6-xCoxO27-δ apatites (x=0.0, x=0.2 and x=0.8) samples calcined at 1000 °C.
